# Aspects of Religious Life as Determinants of the Subjective Health Assessment of Religious Sisters: The Role of Prayer, Community, and Daily Practices

**DOI:** 10.3390/healthcare14050691

**Published:** 2026-03-09

**Authors:** Paulina Teodorczyk, Paweł Najechalski, Maciej Walędziak, Anna Różańska-Walędziak

**Affiliations:** 1 Clinical Nursing Department, Faculty of Medicine, Collegium Medicum, Cardinal Stefan Wyszynski University in Warsaw, 01-938 Warsaw, Poland; s.stellacsfn@gmail.com; 2 Faculty of Medicine, Lazarski University, 02-662 Warsaw, Poland; pawel.najechalski@lazarski.pl; 3 Department of General, Oncological, Metabolic and Thoracic Surgery, Military Institute of Medicine–National Research Institute, Szaserów 128 St., 04-141 Warsaw, Poland; 4 Department of Human Physiology and Pathophysiology, Faculty of Medicine, Collegium Medicum, Cardinal Stefan Wyszynski University in Warsaw, 01-938 Warsaw, Poland; aniaroza@tlen.pl

**Keywords:** health attitudes, religious community, healthy lifestyle, interpersonal relationships

## Abstract

Introduction: Religious practices can shape lifestyles, influence health choices, and help individuals cope with illness and suffering. Understanding which aspects of religiosity support health-promoting attitudes is particularly important. This study explores how belonging to a religious community affects health and well-being among religious sisters. Materials and Methods: An anonymous survey was conducted among 463 women from international, apostolic Catholic congregations in Poland and 33 other countries. The questionnaire included questions on lifestyle, physical health (including diet, physical activity, sleep, chronic conditions, and medication use), and perceptions of how community life influences health and encourages health-conscious behaviors. Results: Overall, 57% of participants reported following a healthy lifestyle, most commonly sisters aged 65 and older (73%). Non-Polish sisters and those living outside Poland were more likely to report healthy habits. Among sisters who saw their community as beneficial for health, 69% led a healthy lifestyle. Retreats, a sense of belonging, communal prayers, and vacations were consistently rated as having the most positive impact on well-being, particularly among older sisters and missionaries. Conclusions: Life in a religious community appears to support health both directly, through structured daily routines and shared responsibilities, and indirectly, by providing social support and fostering a sense of purpose. Spiritual practices, rest, and close interpersonal relationships emerge as the most influential factors for well-being, while formal obligations such as wearing religious attire or attending formation meetings were rated as less impactful. These findings highlight the important role of communal life in promoting both physical and spiritual health among religious sisters.

## 1. Introduction

Religion is an important determinant of health at both individual and societal levels. The literature on the subject increasingly emphasizes the link between religious practices and various health indicators, including both mental and physical well-being. Although these correlations are evident, the mechanisms by which they operate remain unclear and require in-depth, interdisciplinary empirical research. It is essential to identify the elements of religiosity that promote health-conscious attitudes and behaviors, especially considering that approximately 84% of the global population identifies with a religious tradition [1,2,3]. Therefore, religious practices—as an element of everyday life for a large part of society—may significantly influence lifestyles, health choices and ways of coping with illness and suffering. Viewing religion as a determinant of health provides an opportunity to critically examine how religious institutions and spirituality influence social health disparities, contribute to health promotion, and offer social support. It encourages exploration into how these factors intersect to either alleviate or exacerbate health inequalities within communities [4,5].

Consecrated life in the Catholic Church is founded on the evangelical counsels of chastity, poverty, and obedience. Members live communally, dedicating themselves fully to God in imitation of Christ. These voluntary commitments shape a permanent form of Christian life oriented toward spiritual growth, service, and the pursuit of perfect love in the Kingdom of God. In this context, life within a religious community may be conceptualized as an environmental lifestyle variable that structures daily practices, social relationships, and health-related behaviors [6,7]. While religion broadly influences health outcomes, a particularly structured and underexplored context is consecrated religious life within Catholic communities.

The religious life of the lay faithful and that of consecrated persons, although rooted in the same baptism and the same faith, is expressed in distinct forms of daily living.

The teaching of the Second Vatican Council, especially the decree *Perfectae caritatis*, emphasizes that the essence of consecrated life consists in the faithful following of Christ, lived in a spirit of prayer and community. The renewal of this form of life requires both a return to the Gospel and fidelity to the founders’ charism, so that the daily schedule, ascetical practices, and communal relationships become an integral expression of one’s vocation [8].

This perspective is further developed in the apostolic exhortation *Vita consecrata* by John Paul II, which indicates that prayer, community life, and daily fidelity to one’s obligations are not secondary elements, but constitutive dimensions of this path. Consequently, the lifestyle of consecrated persons forms a coherent structure in which community, prayer, and daily practices mutually complement one another [9].

Existing studies, primarily conducted in Poland, suggest that engagement in prayer and transcendence may positively influence psychological well-being and serve as an effective coping strategy in stressful situations. Spirituality is frequently identified as a mediator between religiosity and mental health [10]. Religious formation is also associated with personal maturity, a sense of purpose, and ethical value orientation [10,11]. Scholars have proposed that the lifestyle of religious sisters may represent a cultural alternative to dominant Western models of aging, emphasizing acceptance, relationality, and spiritual depth over productivity and individual “success” [11,12]. The “embracing age” model, developed from research in religious communities, highlights elements such as acceptance of aging, the value of communal life, human dignity independent of productivity, and spirituality as a resource for coping with loss [13]. Nevertheless, this model should not be uncritically generalized, given the specific structural and cultural conditions of religious life [14,15,16]. Critiques of the traditional “successful aging” paradigm further underscore the importance of social relationships, intergenerational interaction, and a sense of being needed, alongside favorable health indicators [17,18,19]. However, previous studies have focused primarily on psychological well-being and conceptual models of aging, while comprehensive quantitative analyses integrating spiritual, communal, and lifestyle dimensions in an international sample of consecrated women remain scarce.

The results presented are part of a study which aimed to examine the health context of religious communities in different countries around the world. For the first time, individual elements of life in a religious community have been identified, used in research, and evaluated by community members for their impact on health. These elements of community life were divided into four domains: spirituality, formal-structural functioning, relational and supportive dimensions, and rest. Spiritual factors include daily prayer in community and regular retreats, while formal factors include maintaining enclosure, wearing religious garb and living in the same house with other sisters [7,20]. Elements related to the functioning of the community include the daily rhythm and duties carried out in accordance with the mission of the institute [21]. Support-related elements include relationships, a sense of belonging, and leisure activities organized within the community [22,23].

This multidimensional categorization corresponds to broader theoretical models of well-being. This study draws conceptually on the Flourishing Framework proposed by VanderWeele, which defines flourishing as a multidimensional state of complete well-being encompassing physical and mental health, meaning and purpose, character and virtue, close social relationships, and financial and material stability. According to this model, human flourishing depends not only on individual factors but also on social and spiritual contexts that nurture purpose and belonging. Religious life within a community provides a distinctive setting in which these dimensions interact daily through shared prayer, service, and interpersonal support. Communal practices among religious sisters may thus enhance key aspects of flourishing—particularly meaning in life and social connectedness—which, in turn, promote better self-rated health and well-being. The current study applies this framework to examine which elements of communal religious life most strongly influence the perception of health among women in consecrated life [24].

Within religious communities, lifestyle is structured and regulated, which makes it a particularly relevant factor in understanding health outcomes. In this context, lifestyle emerges as a central factor, as it is both the most significant and the most modifiable determinant of quality of life, overall health status, and susceptibility to non-communicable diseases (NCDs) [18,19,20]. According to the WHO, NCDs account for 60% of deaths worldwide and 72% in Poland, representing a substantial proportion of healthcare expenditures in industrialized countries [21,22]. Lifestyle is also one of the three main contributors to the rising prevalence of chronic diseases in Western societies. In addition to risky behaviors and unhealthy habits, social and environmental determinants significantly shape population health outcomes. Increasing life expectancy further contributes to a growing number of older adults living with one or more chronic conditions [23].

Maintaining a healthy lifestyle across the life course is therefore essential, as habits formed early in life strongly influence health and well-being in later years. Among older adults, pro-health behaviors are associated with higher education, fewer chronic diseases, absence of falls, adequate sleep, and engagement in physical, social, and cognitive activities [24,25].

The primary aim of the study was to identify which domains of communal religious life are most strongly associated with self-rated health among consecrated women.

This study endeavors to demonstrate the impact of community belonging on health outcomes. The findings of this study could be instrumental in enhancing the lives of individuals within these communities, as well as the overall well-being of communities, particularly in the context of an ageing population.

## 2. Materials and Methods

The presented results constitute a segment of a more extensive study, the objective of which is to ascertain the impact of life in a religious community on the health and well-being of an individual [25]. The data was collected using an anonymous survey, which was disseminated to female, international, apostolic religious congregations in consultation with the Conference of Higher Major Superiors of Polish Religious Congregations. The data collection period extended from April to June 2024.

The study employed a self-developed questionnaire created by the research team based on their clinical experience and prior studies on health and lifestyle. The content validity of the items was verified through expert consultations in both Polish and English versions.

A pilot study with 27 members of various institutes of consecrated life was conducted to test clarity and relevance. Participants suggested adding the response option “several times a year” and making minor stylistic adjustments, which were implemented [25].

The instrument demonstrated acceptable internal consistency (Cronbach’s α = 0.733). Comparative analysis with existing lifestyle studies supported its construct validity. To the best of the author’s knowledge, this is the first tool to distinguish elements of religious life and assess their impact on self-rated health among religious sisters. Participants in the pilot study were not included in the main sample.

The inclusion criteria were membership in a Catholic religious congregation operating under papal law and voluntary consent to participate in the study. A preliminary study was conducted among sisters from congregations who did not subsequently participate in the main survey.

The survey was distributed to 108 religious communities, of which 79 provided responses, yielding a response rate of 73%. However, it was not possible to estimate the number of individual recipients who were reached by the survey, as distribution of the research instrument within each community was contingent upon the individuals responsible for its dissemination.

The questionnaire consisted of three sections. The first part contained demographic information and background characteristics (age, nationality, country of ministry, duration of ministry). The second part focused on lifestyle and physical health, inquiring about eating habits, physical activity, sleep quality, the presence of chronic diseases, and medication use. The third section explored attitudes toward health and examined how community life influences health outcomes and the development of health-conscious behaviors.

All data were analyzed using IBM SPSS Statistics version 25. The statistical significance level was set at *p* = 0.05. The analyses were performed on quantitative and ordinal data derived from Likert-type scales from 1 to 5, where higher scores reflected stronger agreement or more positive assessments of specific aspects of communal and individual health-related behaviors. Descriptive statistics were calculated for all variables, including arithmetic mean, median, standard deviation (SD), and interquartile range (IQR). The normality of the distribution of continuous variables (e.g., age) was verified using the Lilliefors and Shapiro–Wilk tests. To assess relationships between categorical or ordinal variables, the chi-square (χ^2^) test for independence the Pearson correlation coefficient (r) were employed. Partial correlations were also used to examine relationships while controlling for the potential confounding effect of age. To explore the interdependence between perception of a healthy lifestylen factors best predict individual and communal well-being, a series of linear regression models were constructed. Each regression model was preceded by an ANOVA (analysis of variance) test to verify the overall statistical significance of the model. Both regression models utilized standardized beta coefficients (β) to assess the strength and direction of influence.

Ethical Considerations

This study was conducted anonymously and in accordance with the ethical standards outlined in the 1964 Declaration of Helsinki and its subsequent amendments (Fortaleza). Participants were informed about the objective of the study, and informed consent was obtained from each participant. The study was approved by the Bioethics Committee of the National Medical Institute of the Ministry of the Interior and Administration in Warsaw, Poland, with code 25/2024, on 19 April 2024.

## 3. Results

A total of 463 nuns took part in the study. On average, the women were 51.9 years old (with a standard deviation of 14.1 years), and the median age was 50 years. On average, the participants had lived in the community for 30.6 years before the study. There were three age groups: 49% were 49 or younger, 33% were between 50 and 64, and 18% were 65 or older.

The nuns came from 22 countries and worked in 34 countries. They were divided into two groups according to nationality (Polish and others) and according to the country where they ministered, Poland, mission countries and non-mission countries, according to respected criteria of the Congregation for the Evangelisation of Peoples [26]. Mission countries are characterized by a low number of Catholics, a lack of local vocations, and limited financial resources, which affects the lower socio-economic status and charitable activities of religious communities. Non-mission countries include the USA, Europe, Israel, and Australia, while mission countries include Asia, Eastern Europe, Africa, and South America. The youngest participants worked in Poland more often (58%), while the older ones worked in non-mission countries more often (70%). The detailed figures are shown in Table 1.

**Figure 1:**
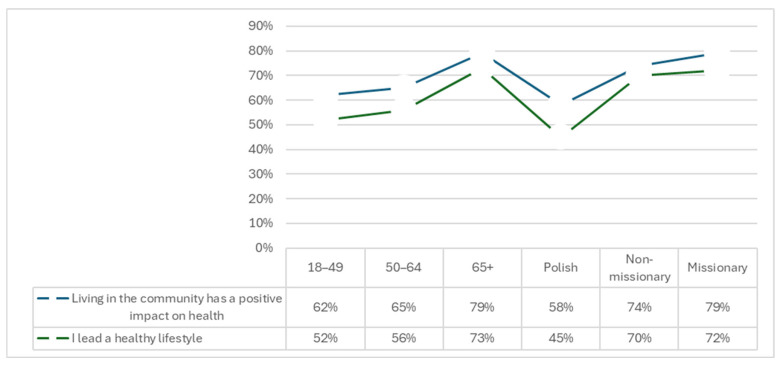
Healthy lifestyle and the positive impact of life in a religious community on health.

A healthy lifestyle was declared by 57% of the participants (*n* = 264), most often by sisters over the age of 65 (73%), which correlates with previous results on daily habits (*p* < 0.05). A significantly higher proportion of sisters of non-Polish nationalities and those residing outside Poland declared a healthy lifestyle (*p* < 0.001). Furthermore, 66% of respondents (*n* = 307) expressed the sentiment that residing within a religious community had a favorable influence on their well-being. Conversely, 25% of respondents (*n* = 118) reported no impact, while 9% (*n* = 38) indicated a negative impact. Statistically significant correlations (*p* < 0.05) concerning age and country of service were noted. The most positive opinions were expressed by sisters working in missionary countries and those over 65 (79% each). Conversely, the most negative assessments were attributed to sisters from Poland (12%) and those in the 18–50 age group (10%), of whom a third could not explicitly assess the community’s impact on health. Notably, none of the sisters who engaged in missionary work reported a negative impact on community life. Further data is displayed in Figure 1.

The majority of respondents expressed a favorable response to all aspects of community life. The element that received the highest number of positive responses was retreats (453). Holidays (445) and communal prayers (439) were the aspects of community life that received the most positive feedback, while the aspects that received the most negative feedback were duties (37), forms of rest and recreation in the community (36), and religious garb (34). Among those who perceive community life as beneficial to their health, the highest number of positive responses was for retreats (302) vacation (299 positive responses), communal prayers (297), the sense of community (295), and interpersonal relationships (293) all received similarly elevated positive ratings. Conversely, the religious habit received the most ‘No effect’ answers (47) and relatively the fewest positive answers (247). Maintaining privacy in cloister also received a significant number of ‘Difficult to say’ answers (36).

A study was conducted to determine the extent to which a set of predictive variables, representing distinct aspects of community life, could account for the variability of the dependent variable—namely, the positive impact of community on health. To address this research objective, an analysis of variance (ANOVA) was performed and the resulting regression model was found to be statistically significant.

Multiple regression analysis indicated that the model significantly predicted the extent to which community life positively influences health, F(13, 432) = 21.369, *p* < 0.001, accounting for approximately 39% of the variance in this outcome (R^2^ = 0.391).

Among the predictors examined, several had a significant positive impact on perceived health: cohabitation with other sisters (B = 0.322, β = 0.279, t = 5.225, *p* < 0.001), quality of interpersonal relationships within the community (B = 0.222, β = 0.204, t = 3.285, *p* = 0.001), the community’s daily rhythm (B = 0.163, β = 0.143, t = 2.917, *p* = 0.004), and fulfillment of duties (B = 0.110, β = 0.098, t = 2.322, *p* = 0.021).

In contrast, strict observance of the cloistered life (B = −0.176, β = −0.150, t = −3.169, *p* = 0.002) and leave (B = −0.153, β = −0.091, t = −2.135, *p* = 0.033) were associated with a significant negative impact on perceived well-being. Table 2 contains all the detailed data.

A linear regression analysis was performed to determine which elements of community life contribute to self-reported engagement in a healthy lifestyle. Linear regression analysis indicated that the model significantly predicted the declaration of a healthy lifestyle, F(13, 432) = 4.984, *p* < 0.001, accounting for 13% of the variance in this outcome (R^2^ = 0.13).

Two variables had a significant positive effect on self-reported health-promoting behaviors: fulfillment of duties (B = 0.165, β = 0.139, t = 2.756, *p* = 0.006) and days of responsibility for prayer (B = 0.166, β = 0.116, t = 2.418, *p* = 0.016). Conversely, observance of the cloistered life exerted a negative influence (B = −0.153, β = −0.122, t = −2.168, *p* = 0.031).

The variables of vacation (*p* = 0.059) and retreats (*p* = 0.074) approached the threshold of statistical significance. Other factors—such as interpersonal relationships, the community’s daily rhythm, formation meetings, and forms of recreation—did not show significant associations with a healthy lifestyle.

## 4. Discussion

The impact of life in a religious community and religious practices on health

The impact of religion on health has been a subject of interest in recent research. The present study’s findings demonstrate a discernible positive correlation between the positive evaluation of the impact of community life on health and the declared pursuit of a healthy lifestyle. Individuals who perceive community as a health-promoting factor are likelier to adopt health-promoting behaviors. This finding suggests that life in a religious community is important as a source of social support and a motivational factor conducive to maintaining healthy habits and lifestyles. A comprehensive review of the extant biomedical literature has indicated that aspects related to religion and spirituality can exert a positive or negative influence on health, with the impact at the individual level appearing to be positive [2]. A substantial corpus of research has demonstrated that individuals who belong to religious communities exhibit considerably enhanced levels of well-being. These outcomes encompass a substantially diminished risk of cardiovascular and cancer mortality among female members, a six-fold reduction in the incidence of suicides, and a significantly mitigated risk of depression [27,28]. Participation in religious practices has been demonstrated to enhance health-promoting behaviors, including smoking cessation and the adoption of a later age of first smoking initiation, accompanied by a reduction in the consumption of illegal substances [29,30]. Moreover, research has demonstrated that engagement in religious communities can lead to flourishing in several areas of human life, including social relationships, mental and physical health, and life satisfaction [24,31].

Self-assessment of health in relation to age

In our study, older adults exhibited the most positive perception of their own health and the impact of the community on health. This is likely attributable to how the community has, over the years, cultivated positive attitudes among its members, thereby facilitating positive aging. This assertion is corroborated by the research of McManus, who found that daily involvement in religious and spiritual practices, meditation, complacency, and a positive attitude towards successful aging, acceptance of life, a sense of faith, and a positive attitude towards the afterlife influence individuals [20]. Two seminal studies in geriatrics and gerontology, known as the Nun Study and the Religious Order Study, substantiate the protective effect of belonging to a religious community and participating regularly in religious and cognitive activities against Alzheimer’s disease [32,33,34].

Impact elements of religious life on health

Notably, most responses to all elements of community life are positive, suggesting a consistent evaluation of the various aspects of religious life among individuals who perceive a favorable influence of the community on their health. An ethnographic study by Amiiotte et al. found that a religious community significantly impacts an individual’s daily life, providing a framework based on prayer and spiritual practices. The lifestyle of a community member is transformed by the necessity to subordinate oneself to shared values, which involves giving up a certain degree of individual autonomy. Therefore, personal talents and preferences are embedded in a community context and evaluated regarding their compatibility with the collective ideal. Consequently, the daily life of a monastery member becomes a space of continuous self-improvement, in which the practice of obedience, humility, and adaptation serves to maintain the harmony of the community and the individual’s spiritual growth [35]. In our research, the most highly rated elements (retreats, holidays, prayers, days of responsibility for prayer) are associated with spirituality and rest. The sisters rate them as having the most positive impact on their well-being. An experimental study was conducted on a group of 33 Roman Catholic nuns who had undergone electroencephalograms (EEGs) during periods of meditation. The study revealed that prayer increased alpha-wave activity in the occipital region of the brain. Furthermore, it was observed that brain activity in the left frontocentral lobe exhibited an age-related increase [36]. In addition, an electroencephalogram (EEG) study of Muslims during prayer revealed that salat reduced parasympathetic activity and decreased sympathetic activity. This finding supports the hypothesis that regular prayer can promote relaxation, reduce anxiety, and decrease cardiovascular risk [37,38]. Conversely, elements that received the lowest ratings, such as religious attire, community recreational activities, and formation meetings, may necessitate consideration for enhancement, as they were perceived to exert a less favorable influence on well-being.

The existence of strong correlations between elements in the ratings further underscores the interconnection between them. For instance, the strong correlation between ‘Interpersonal relationships’ and ‘Sense of community’ indicates that individuals who rate interpersonal relationships positively also tend to rate sense of community. Some studies suggest that belonging to a religious community may have a more substantial effect on an individual’s health than the level of personal religiosity. Nevertheless, active participation in such a community and personal belief significantly impact health more than non-believers [39,40]. The results of our research suggest that spiritual and rest-related aspects are particularly valued, while some formal (e.g., religious garb) and community (e.g., formation meetings) elements are not. Converging conclusions can be drawn in the descriptive study conducted by Palmisano [41]. One of the participants notes that there is a tension between the contemplative and the active elements, which is reflected in the rhythm of the day. The day is divided into two distinct segments: the first is dedicated to work and activity. The second is devoted to community and meditation. This division of activities has been identified as one of the most important challenges of social life, especially in the perspective of the need to introduce changes in order to better respond to the challenges of the modern world [41,42].

Limitations of the study

One of the primary limitations of the survey was attributable to its electronic format and the limited availability of the paper version, which might have resulted in a reduced participation rate among older members of the congregation and those who do not engage with media.

The study may be limited by factors such as recall bias, social desirability bias, and the subjective nature of patient opinions. Nonetheless, there was no incentive for participants to provide dishonest responses.

Although the study has an international focus, most participants are Polish nationals, even when they are stationed in countries outside of Poland. This factor could potentially influence the study’s findings and should be considered when analyzing results in mission countries where the number of local nuns is lower. Therefore, it is essential to interpret the results with an emphasis on the health of the missionary nuns.

The strong representation of Poland in the sample restricts the applicability of the results to other Catholic contexts, particularly in mission countries where the number of local sisters is lower. Furthermore, the absence of a control group weakens the strength and persuasiveness of the conclusions.

The next limitation of the present study is the use of self-rated health as the primary outcome measure. Although self-rated health is a widely accepted and validated indicator in epidemiological research, it remains a subjective measure and may be influenced by individual perceptions, personal expectations, cultural norms, and current emotional states. Consequently, it may not fully reflect objective health status.

## 5. Conclusions

Religious sisters who perceive community as a health-promoting factor are more likely to engage in health-promoting behaviors. This finding suggests that living in a religious community is important as a source of social support and as a motivational factor in maintaining healthy habits and lifestyles.

The consistently positive ratings of all elements of community life suggest that certain aspects of social life—such as leisure (holidays) and retreats, structured rhythms and interpersonal relationships—are related to overall community health and spiritual well-being.

The results suggest that spiritual and restful aspects are particularly valued, while some formal (such as religious dress) and communal (such as training meetings) elements are valued less, and their improvement may contribute to better self-perceived health.

The results suggest that the key factors contributing to the positive impact of the community on health are primarily close interpersonal relationships, communal living, and a structured daily rhythm, whereas excessive strictness due to adherence to the cloister and a lack of time for rest (vacation) may limit perceived well-being.

## Figures and Tables

**Table 1:** Demographic characteristics of participants.

Mean age, years (IQR)		51.9 (21)
Median age, years (SD)		50 (+/− 14.1)
The mean number of years in the congregation (SD)		30.6 (+/− 15.8)
Age n (%)	18–49	228 (49%)
	50–64	151 (33%)
	65+	81 (18%)
Nationality n (%)	Polish	323 (69.8%)
	Other	140 (30.2%)
Country of ministry n (%)	Poland	250 (54%)
	Missionary country	61 (13.2%)
	Non- missionary country	148 (32%)
	No answer	4 (0.8%)

**Table 2:** Multiple regression results for the variable ‘Community positively influences health’.

	Unstandardized Coefficients		Standardized Coefficients	t	*p*
	B	Standard Error	Beta		
(Constant)	0.904	0.467		1.938	0.053
Formation meetings	0.059	0.051	0.058	1.155	0.249
Forms of recreation and rest planned in the community	0.106	0.055	0.101	1.947	0.052
Maintaining privacy of the cloister	−0.176	0.056	−0.150	−3.169	0.002
Communal living	0.322	0.062	0.279	5.225	<0.001
Relationships in the community	0.222	0.068	0.204	3.285	0.001
Sense of connection in the community	−0.031	0.069	−0.027	−0.454	0.650
Religious garb	0.068	0.046	0.073	1.478	0.140
Community rhythm of the day	0.163	0.056	0.143	2.917	0.004
Common prayers	0.009	0.074	0.005	0.116	0.908
Retreats	−0.060	0.089	−0.031	−0.667	0.505
Days of private prayer	0.091	0.054	0.067	1.679	0.094
Duties performed	0.110	0.047	0.098	2.322	0.021
Vacation	−0.153	0.072	−0.091	−2.135	0.033

## Data Availability

The original contributions presented in this study are included in the article. Further inquiries can be directed to the corresponding author.
